# Palliative and End-of-Life Care Utilization in Cardiogenic Shock Complicating Acute Myocardial Infarction: A Population-Based Study

**DOI:** 10.1016/j.jacadv.2026.102869

**Published:** 2026-06-11

**Authors:** Lee H. Sterling, Shannon M. Fernando, Robert Talarico, Danial Qureshi, Pietro Di Santo, Sean van Diepen, Margaret S. Herridge, Susanna Price, Daniel Brodie, Eddy Fan, Dale M. Needham, Damon C. Scales, Alain Combes, Arthur S. Slutsky, Holger Thiele, Benjamin Hibbert, Peter Tanuseputro, Rebecca Mathew

**Affiliations:** a Division of Critical Care Medicine, University of British Columbia, Vancouver, British Columbia, Canada; b CAPITAL Research Group, Division of Cardiology, University of Ottawa Heart Institute, Ottawa, Ontario, Canada; c Department of Critical Care Medicine, Queen's University, Kingston, Ontario, Canada; d Department of Critical Care, Lakeridge Health, Oshawa, Ontario, Canada; e Clinical Epidemiology Program, Ottawa Hospital Research Institute, Ottawa, Ontario, Canada; f ICES, Toronto, Ontario, Canada; g Nuffield Department of Population Health, University of Oxford, Oxford, United Kingdom; h Department of Critical Care Medicine and Division of Cardiology, Department of Medicine, University of Alberta, Edmonton, Alberta, Canada; i Canadian VIGOUR Centre, University of Alberta, Edmonton, Alberta, Canada; j Interdepartmental Division of Critical Care Medicine, University of Toronto, Toronto, Ontario, Canada; k Toronto General Hospital Research Institute, University Health Network, Toronto, Ontario, Canada; l Institute of Health Policy, Management and Evaluation, Dalla Lana School of Public Health, University of Toronto, Toronto, Ontario, Canada; m Departments of Cardiology & Critical Care, Royal Brompton Hospital, National Heart & Lung Institute, Imperial College, London, United Kingdom; n Department of Medicine, Division of Pulmonary and Critical Care Medicine, Johns Hopkins University School of Medicine, Baltimore, Maryland, USA; o Department of Physical Medicine and Rehabilitation, Johns Hopkins University School of Medicine, Baltimore, Maryland, USA; p Department of Critical Care Medicine, Sunnybrook Health Sciences Centre, Toronto, Ontario, Canada; q Sorbonne Université, Institute of Cardiometabolism and Nutrition, Paris, France; r Service de Médecine Intensive-Réanimation, Hôpitaux Universitaires Pitié Salpêtrière, Assistance Publique-Hôpitaux de Paris, Institut de Cardiologie, Paris, France; s Department of Internal Medicine/Cardiology, Heart Center Leipzig at Leipzig University and Leipzig Heart Science, Leipzig, Germany; t Department of Cardiovascular Medicine, Mayo Clinic, Rochester, Minnesota, USA; u Department of Family Medicine and Primary Care, The University of Hong Kong, Hong Kong SAR, China

**Keywords:** cardiogenic shock, cardiology, critical care, myocardial infarction, palliative care

## Abstract

**Background:**

Little is known about end-of-life trajectories in survivors of cardiogenic shock complicating acute myocardial infarction (AMI-CS) who die beyond their index admission, or utilization of palliative care services in AMI-CS survivors.

**Objectives:**

This study aimed to examine long-term palliative and end-of-life care among AMI-CS survivors.

**Methods:**

This was a population-based, retrospective cohort of AMI-CS survivors in Ontario, Canada, from 2009 to 2020 who died during longitudinal follow-up.

**Results:**

We identified 3,881 AMI-CS survivors (2009-2020) who died after discharge and before March 2024. The median survival time was 1,096 days (IQR: 312-2,139 days). Overall, 2,100 patients (54.1%) died in acute care, with no difference between those who did and did not receive palliative care. Patients who did not receive palliative care were more likely to die in intensive care units (ICU) than those who did (23% vs 17%, absolute standard difference 0.15). Most patients received palliative care in the final year of life (n = 2,485, 64%); 1,057 patients (42.5%) had outpatient visits, 505 patients (20.3%) had inpatient palliative care consultations, and 327 patients (13.2%) had palliative care hospitalizations. Palliative care, however, was most commonly initiated in the last 14 days of life (1,185 patients, 47.7%). Earlier palliative care referrals were associated with reduced rates of dying in hospital (adjusted OR: 0.50; 95% CI: 0.42-0.65) and ICU (adjusted OR: 0.34; 95% CI: 0.26-0.45).

**Conclusions:**

Early and intermediate term palliative care involvement was associated with reduced risk of death in hospital and ICU. Such consultation may improve end-of-life outcomes in AMI-CS survivors.

Cardiogenic shock (CS) is a clinical syndrome characterized by systemic hypoperfusion secondary to low cardiac output^1^ and is often caused by acute myocardial infarction leading to left ventricular systolic dysfunction.^2^ CS complicating acute myocardial infarction (AMI-CS) occurs in 3% to 13% of myocardial infarction and confers poor short-term prognosis.^3^ These patients often experience significant morbidity and mortality in the months and years after their initial hospitalization.^4^, ^5^, ^6^ Among AMI-CS survivors, 42% require increased support in care from their preadmission baseline, 47% are readmitted within 1 year, with 15% dying within 1 year.^5^ Survivors of AMI-CS have high health care resource utilization and costs after discharge^7^ and experience significant mental health morbidity.^8^

Although AMI-CS survivors remain at high risk for future morbidity and mortality,^5^^,^^6^ little is known about end-of-life trajectories in AMI-CS survivors who experience death beyond 30 days from their index event.^9^^,^^10^ Similarly, there is a substantial knowledge gap regarding the utilization of palliative care services in AMI-CS. In contrast, end-of-life trajectories have been well described in advanced heart failure patients.^11^ The need to better characterize the use of palliative care and the end-of-life trajectory in AMI-CS was highlighted as a particular American Heart Association (AHA) research priority.^1^ Therefore, we performed a population-level analysis of AMI-CS survivors with the following objectives: 1) to describe the place of death and care settings for AMI-CS decedents in the last month of life; 2) to quantify and describe palliative care service in AMI-CS decedents during their last year of life; 3) to assess resource utilization in AMI-CS decedents; and 4) to identify factors associated with death in acute care hospital and intensive care for AMI-CS decedents.

## Methods

This was a population-based retrospective cohort study using health administrative databases housed at ICES (formerly known as the Institute for Clinical Evaluative Sciences) in the province of Ontario, Canada (Supplemental Table 1). Ontario has a population of 15.6 million and a single-payer health care system where all medically necessary health care services, physician, hospital, and demographic information from residents are captured in a series of individually linked provincial databases. These databases are stored and linked at ICES, an independent, nonprofit custodian of health data. ICES is funded via an annual grant from the Ontario Ministry of Health. Encrypted health card numbers are used as unique identifiers and linked across several different health administrative databases at ICES. Studies utilizing administrative data at ICES fall under section 45 of the Personal Health Information Protection Act of Ontario and do not require research ethics board approval. Data were thus collected without requiring individual patient consent. We adhered to the Strengthening the Reporting of Observational Studies in Epidemiology guidelines for reporting observational studies (Supplemental Table 2).^12^

### Study design

We identified consecutive adult patients with AMI-CS using criteria described previously.^5^^,^^8^ Briefly, patients meeting all the following were included: 1) ≥18 years of age; 2) admitted with AMI-CS to an intensive care unit (ICU) between January 1, 2009, and December 31, 2020; 3) discharged from hospital following their index admission; 4) died between hospital discharge and March 31, 2024. Patients with AMI were identified through the Discharge Abstract Database using previously established algorithms in conjunction with the Ontario Myocardial Infarction Dataset which identify AMI with 92% to 100% accuracy.^13^ Given the administrative nature of our data set, we were unable to stratify patients with AMI-CS by Society for Cardiovascular Angiography & Interventions shock stages.^14^ Instead, we inferred the presence of shock using the following combined criteria: 1) admission to an ICU, identified using validated algorithms;^15^ 2) treatment with vasoactive medications (in keeping with guideline-directed care for patients with CS);^16^ and 3) evidence of end-organ hypoperfusion using data from the Critical Care Information System database, using previously described methods.^5^ Those presenting with out-of-hospital cardiac arrest were excluded in keeping with previous trials of AMI-CS patients.^17^^,^^18^

Relevant patient comorbidities using hospitalization and physician billing in the 5 years prior to the index hospitalization, clinical details including median Multi Organ Dysfunction Score at admission, palliative care in the year prior to an index AMI-CS admission, total ICU and hospital length of stay, number of days requiring vasoactive medications, and ICU interventions including mechanical ventilation, renal replacement therapy, and mechanical circulatory support were collected (Supplemental Table 2). Wherever possible, we used previously validated algorithms to identify comorbidities and hospital interventions (Supplemental Table 3).^19^^,^^20^

### Outcome Measures

Our primary outcome measure was place of death for AMI-CS survivors as this is a key marker of high quality end of life care.^21^ To determine place of death, we identified the care setting (as detailed below) where the discharge disposition indicated death and the discharge date concurred with the death date recorded in the Registered Persons Database, thus indicating that the individual died in that care setting. Ontario uses mandatory reporting for all deaths, which are centrally held in the Registered Persons Database with data until March 31, 2024 inclusive.

We examined several secondary outcomes. First, care settings, defined as length of stay in days in a particular setting in the last month of life. Care settings were defined as one of the following based on a previously described hierarchy: 1) ICU; 2) non-ICU acute care (which included admissions into same-day surgery and ambulatory care); 3) palliative care unit, which also includes acute care admissions during which palliative care was the most responsible service provider; 4) subacute care, which encompassed complex continuing care, rehabilitation services, and/or mental health services; 5) long-term care; and 6) community, which includes all other settings.^22^

Second, we quantified the use of palliative care services for AMI-CS survivors. We identified the initiation and utilization of palliative care services during the index hospital stay and after discharge in AMI-CS survivors by identifying claims from physician-based palliative care billing codes within institutions such as hospitals, emergency departments, long-term care and complex continuing care homes, and palliative home services using previously published methods (Supplemental Table 4).^23^ To capture a broad spectrum of physician-delivered care, this definition encompassed both billing claims from palliative care specialists and palliative care visit codes from generalist physicians. The setting of palliative care delivery was further characterized as occurring in the community (ie, private home, retirement homes, or hospices) or within institutional care (ie, during acute care hospitalization, complex continuing care, long-term care, or the emergency department). The timing of the initiation of palliative care was calculated by subtracting an individual’s date of death from the date of palliative care initiation. Patients were categorized into the following groups, according to initiation time before death: early (≥60 days), intermediate (≥15 to <60 days), late (≥0 to ≤14 days), and never (no initiation), based on previously published timeframes.^24^ A minority of patients with advanced heart failure with poor short-term prognosis are referred for advanced therapies such as durable left ventricular assist device (LVAD) implant or heart transplant rather than palliative care. We initially planned to identify rates of LVAD implant and heart transplant in AMI-CS survivors but the overall number of durable LVADs and heart transplantation in our cohort was too small to report due to ICES small cell size rules.

Third, we performed a cost analysis for AMI-CS survivors in the last month of life. Costs were divided into acute care, continuing care, and patient sectors based on previously defined methods.^7^ Acute care sectors included all inpatient care and emergency departments. Continuing care sectors included complex-continuing care, long-term care, inpatient rehabilitation, and home care. Outpatient care sectors included outpatient clinics, laboratory investigations, and drugs. Direct cost billings and case-mix methodology were used to determine cost data associated with sectors that use global budgets.^7^ As well, we compared costs between those who did and did not receive palliative care services in the last year of life. All costs are reported in February 2025 U.S. dollars.

### Statistical analysis

Data are presented as mean values, with SDs, or medians, with IQR, where appropriate. Between-group differences are expressed using absolute standardized differences (ASD), with an ASD >0.1 representing a significant between-group difference. To determine statistical significance in trends, the Cochran-Armitage test for linear trend in proportions and a one-way analysis of variance for linear trend in means were used, with a *P* value <0.05 being considered significant. Finally, we graphed place of care trajectories of AMI-CS survivors, showing the number of people in a particular setting on a day-by-day basis within the last 4 weeks of life.

To identify factors associated with the important patient-centered outcomes of dying in hospital and dying in ICU, we fit a multivariable logistic regression model and report ORs with corresponding 95% CIs. In accordance with the prognosis research strategy framework, we identified model variables a priori.^25^ To ensure that the explanatory variables occurred before the outcome, we limited out multivariable regression to outcomes that were terminal rather than other variables such as receipt of palliative care. Data were complete save for minimal missing data for geographic variables (neighborhood income quintile, rurality) for which only 0.4% had missing values. These were missing completely at random, and for relevant regression modeling, we imputed the most common value (urban neighborhood and income quintile 3).

All statistical analyses were conducted using SAS Enterprise Guide 7.1 (SAS Institute, Inc).

## Results

We identified 3,881 AMI-CS survivors who died after discharge, and prior to March 31, 2024. Baseline characteristics and index admission characteristics are shown in Table 1. Overall, 2,526 (65.1%) of patients were male and mean age at index admission was 73.2 ± 11.1 years. The majority of patients resided in an urban setting (85.8%) and were at home living independently (77.6%) prior to their index admission. The most common comorbidities were hypertension (57.9%) and diabetes mellitus (51.4%). Median (IQR) ICU and hospital length of stay was 6^4^, ^5^, ^6^, ^7^, ^8^, ^9^, ^10^ and 15^9^, ^10^, ^11^, ^12^, ^13^, ^14^, ^15^, ^16^, ^17^, ^18^, ^19^, ^20^, ^21^, ^22^, ^23^, ^24^, ^25^, ^26^ days, respectively. Palliative care utilization in the year prior to the index AMI-CS admission occurred in 168 (4.33%) patients. During the index hospital stay, only 34 (0.5%) patients had a palliative care interaction.

**Table 1:** Baseline Demographics and Index Hospitalization Characteristics of Patients With Cardiogenic Shock Complicating Acute Myocardial Infarction in Ontario, 2009-2020 (N = 3,881)

Sex – n (%)	
Female	1,355 (34.9)
Male	2,526 (65.1)
Age – y - mean ± SD	73.2 ± 11.1
Income quintile – n (%)	
Lowest	964 (24.8)
Low	807 (20.8)
Middle	762 (19.6)
High	742 (19.1)
Highest	589 (15.2)
Unknown	17 (0.4)
Rurality – n (%)	
Rural	545 (14.0)
Urban	3,330 (85.8)
Comorbidities – n (%)	
Hypertension	2,249 (57.9)
Diabetes	1993 (51.4)
Renal failure	1,045 (26.9)
Cancer	961 (24.8)
Prior congestive heart failure	910 (23.4)
COPD	502 (12.9)
Dyslipidemia	432 (11.1)
Atrial fibrillation/flutter	318 (8.2)
Stroke	176 (4.5)
Cirrhosis	46 (1.2)
Charlson Index ≤2 – n (%)	3,244 (83.6)
Length of index hospital stay – d – median (IQR)	
ICU	6 (3-10)
Total	15 (5-22)
MODS score at ICU admission - median (IQR)	4 (3-6)
ICU d on vasoactive infusion - median (IQR)	2 (1-4)
Invasive mechanical ventilation – n (%)	3,622 (52.9)
Renal replacement therapy – n (%)	1,273 (18.6)
Mechanical circulatory support – n (%)	1,052 (15.4)
Palliative services in the year prior to admission – n (%)	168 (4.33)
Palliative services during index admission – n (%)	34 (0.5)

AMI-CS = cardiogenic shock complicating acute myocardial infarction; COPD = chronic obstructive pulmonary disorder; ICU = intensive care unit; MODS = Multi Organ Dysfunction Score.

### Place of death and care settings

The median (IQR) survival time after the index AMI-CS admission was 1,096 (312-2,139) days. Place of death is shown in Table 2, stratified between patients who did and did not receive palliative care in the last year of life. In total, 2,100 patients (54%) died in acute care (Table 2), with no difference between those who did and did not receive palliative care (54.2% vs 54% vs, ASD 0). Patients who did not receive palliative care were more likely to die in ICU than patients who did (23% vs 17%, ASD 0.15). Trends in places of care and hospital admissions in the last 30 days of life are shown in the Central Illustration. Overall survival curves are shown in Supplemental Figure 1.

**Table 2:** Places of Care in Last 30 Days of Life and Place of Death in Survivors of Cardiogenic Shock Complicating Acute Myocardial Infarction

	AMI-CS Patients With Palliative Care in Last Year of Life (n = 2,485)	AMI-CS Patients With No Palliative Care in Last Year of Life (n = 1,396)	ASD
ICU admission in last 30 d of life - n (%)	731 (29.4)	465 (33.3)	0.08
Days spent in different locations in the last 30 d of life – median (IQR)			
ICU	0 (0-2)	0 (0-2)	0.06
Acute care hospitalization (excluding ICU)	8 (2-18)	3 (0-11)	0.44
Subacute care	0 (0-0)	0 (0-0)	0.2
Long-term care facility	0 (0-0)	0 (0-0)	0.08
Home/community	14 (0-25)	24 (5-29)	0.44
Place of death - n (%)			
Non-ICU acute care	920 (37.0)	436 (31.2)	0
ICU	423 (17.0)	321 (23.0)	0.15
Subacute care	266 (10.7)	55 (3.9)	0.26
Long-term care facility	226 (9.1)	126 (9.0)	0
Palliative care facility	147 (5.9)	0 (0.0)	0.35
Home/community	503 (20.2)	458 (32.8)	0.29

ASD = absolute standardized differences; other abbreviations as in Table 1.

**Figure 1:**
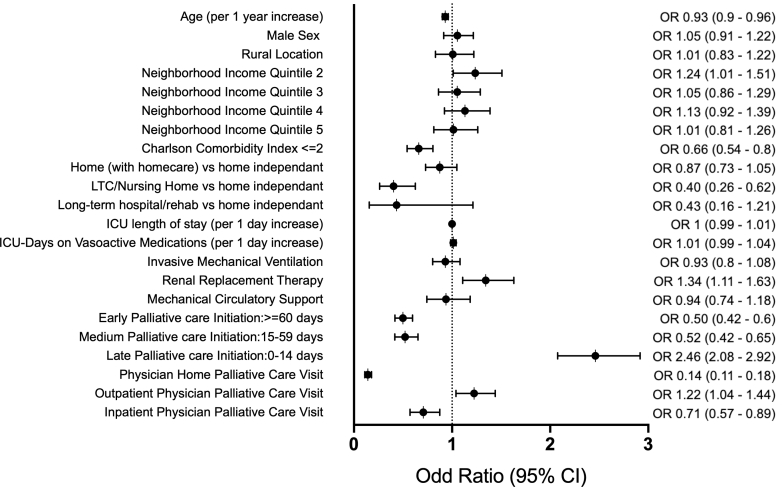
Factors Associated with Dying in Hospital Forest plot of multivariable logistic regression analysis of factors associated with dying in acute care hospital in survivors of cardiogenic shock complicating acute myocardial infarction. ICU = intensive care unit; LTC = long term care.

**Figure 2:**
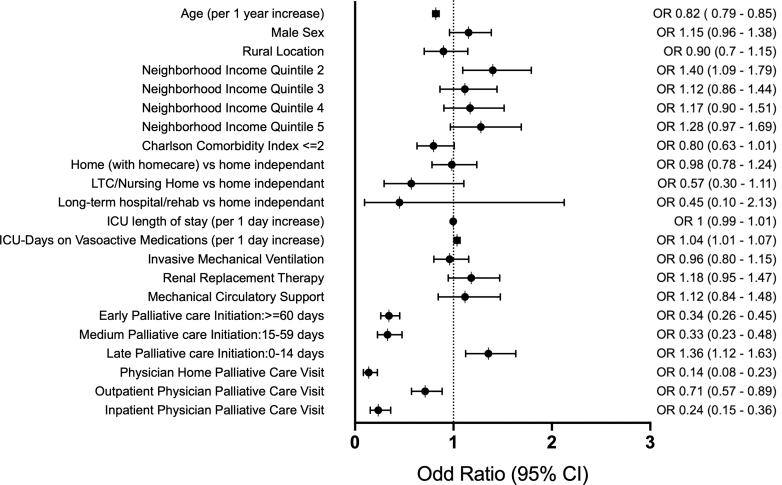
Factors Associated with Dying in Intensive Care Unit Forest plot of multivariable logistic regression analysis of factors associated with dying in ICU in survivors of cardiogenic shock complicating acute myocardial infarction. Abbreviation as in Figure 1.

**Central Illustration:**
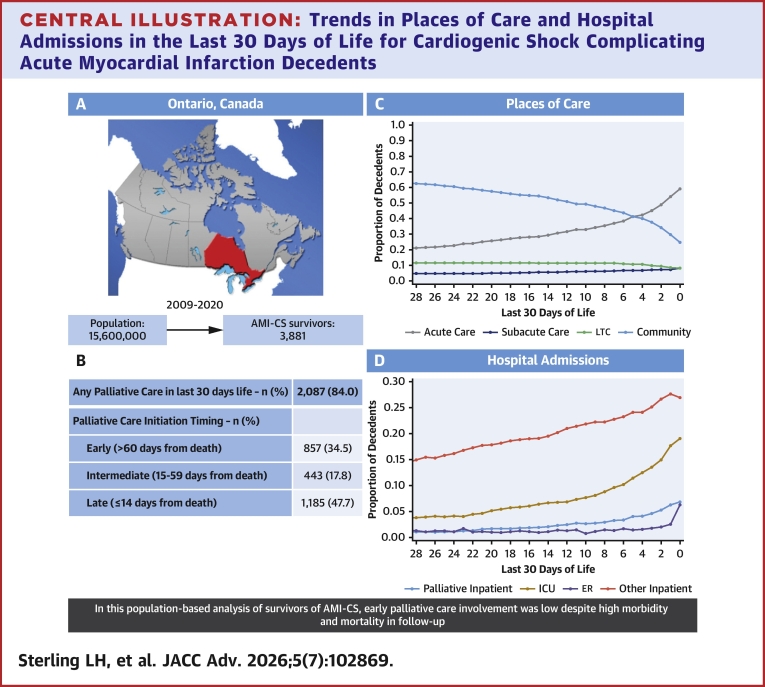
Trends in Places of Care and Hospital Admissions in the Last 30 Days of Life for Cardiogenic Shock Complicating Acute Myocardial Infarction Decedents (A) This population-based retrospective cohort study was performed in the province of Ontario, Canada (population 15,600,000) and identified 3,881 patients who survived an admission with cardiogenic shock complicating acute myocardial infarction who went on to die during follow-up. (B) Details of the timing of palliative care involvement in cardiogenic shock complicating acute myocardial infarction survivors in the last year of life are shown. Palliative care was most commonly initiated in the last 14 days of life. (C) Trends in places of care in the last 30 days of life for cardiogenic shock complicating acute myocardial infarction decedents are shown. In the last 30 days of life, there was a sharp increase in admissions to acute care and in total 2,100 patients (54%) died in acute care. (D) Trends in hospital admissions in the last 30 days of life for cardiogenic shock complicating acute myocardial infarction decedents are shown. When admitted to hospital, patients were most often admitted to acute inpatient units with a steady increase in ICU admissions in the last days of life. AMI-CS = cardiogenic shock complicating acute myocardial infarction.

Places of care in the last 30 days of life are shown in Table 2. Patients who received palliative care in the last year of life spent more days (median [IQR]) in acute care (8 [2-18] days vs 3 [0-11] days, ASD 0.44) and less time in the community (14 [0-25] days vs 24 [5-29] days, ASD 0.44) than those who did not. Mean number of ICU days and days spent in subacute care and long-term care were not significantly different between those who did and did not receive palliative care.

### Palliative care delivery

Details of palliative care delivery are shown in Table 3. The majority of AMI-CS survivors received palliative care in the final year of life (2,485 patients, 64.0%). Palliative care was most commonly initiated in the last 14 days of life (1,185 patients, 47.7%). Of these, 1,057 patients (42.5%) had an outpatient palliative care visit with a mean 1.3 ± 2.7 visits. In contrast, 443 patients (17.8%) had home palliative care visits, and 509 patients (20.5%) had home palliative care services initiated; 505 patients (20.3%) had inpatient palliative care consultation, and 327 patients (13.2%) had palliative care hospitalizations. Non-palliative home care services were utilized in both patients who did and did not receive palliative care but were more commonly utilized by patients receiving palliative care (1,160 patients, 46.7% vs 556 patients, 39.8%, ASD 0.14).

**Table 3:** Palliative Care Delivery and Services Received in Last Year of Life in Cardiogenic Shock Complicating Acute Myocardial Infarction (N = 2,485)

Any palliative care in last 30 d life – n (%)	2087 (84.0)
Palliative care initiation timing – n (%)	
Early (>60 d from death)	857 (34.5)
Intermediate (15-59 d from death)	443 (17.8)
Late (≤14 d from death)	1,185 (47.7)
Outpatient palliative physician visit – n (%)	1,057 (42.5)
Number palliative outpatient visits	1.3 ± 2.7
Home palliative physician visit – n (%)	443 (17.8)
Number palliative home visits	0.5 ± 1.6
Home palliative care services – n (%)	509 (20.5)
Number home palliative service visits	2.5 ± 6.6
Home non-palliative care services – n (%)	1,160 (46.7)
Number home non-palliative service visits	4.2 ± 7.3
Inpatient palliative physician visits – n (%)	505 (20.3)
Number inpatient palliative physician visits	0.7 ± 2.4
Inpatient palliative care – n (%)	327 (13.2)

Abbreviation as in Table 1.

Values are median (IQR) unless otherwise specified.

### Cost analysis in the last 30 days of life

Full details on cost breakdown across all sectors and between patients who received palliative care vs those who did not are shown in Table 4. Mean (SD) total cost among all patients was $18,405 ± $19,288 in the last year of life. Mean (SD) costs across all patients for inpatient care, emergency department care, outpatient clinics, and home care were $12,075 ± $17,318, $442 ± $444, $379 ± $759, and $756 ± $1,398, respectively.

**Table 4:** Cost Analysis in Last 30 Days of Life for Survivors of Cardiogenic Shock Complicating Acute Myocardial Infarction

	AMI-CS Patient With Palliative Care in Last year of Life (n = 2,485)	AMI-CS Patient With No Palliative Care in Last year of Life (n = 1,396)	Mean Difference (95% CI)
Total costs	20,086 ± 19,296	15,410 ± 18,914	4,676 (3,427-5,926)
Acute care sectors			
Inpatient	13,246 ± 17,652	9,989 ± 16,509	3,258 (2,148-4,368)
ED	464 ± 449	404 ± 433	60 (31-78)
Continuing care sectors			
Complex continuing care	922 ± 3,818	581 ± 2,858	341 (146-537)
Long-term care	482 ± 1,209	401 ± 1,113	81 (6-157)
Rehabilitation	207 ± 1,823	314 ± 2,208	−107 (−243-30)
Home care	939 ± 1,612	430 ± 805	508 (432-584)
Outpatient care			
Outpatient clinics	479 ± 870	202 ± 402	277 (236-317)
Laboratory (OHIP)	15 ± 40	17 ± 38	−1 (−4 to 1)
Drugs	281 ± 603	261 ± 846	20 (−31-70)
Physician billings	2,381 ± 2,843	1960 ± 2,547	422 (248-596)

ED = emergency department; OHIP = Ontario Health Insurance Plan; other abbreviation as in Table 1.

All values expressed as U.S. dollars in February 2025.

AMI-CS patients who had palliative care in the last year of life had higher total resource utilization vs those who did not ($20,0867 ± $19,296 vs $15,410 ± $18,914; mean difference (95% CI) $4,676 ($3,427 – $5,926)). This was driven by increased inpatient ($13,246 ± $ 17,652 vs $ 9,989 ± $16,509; mean difference [95% CI] $3,258 [$2,148 – $4,368]), emergency department ($464 ± 449 vs $404 ± 433; mean difference [95% CI] $60 [$31 – $78]), and complex continuing care ($922 ± 3,818 vs $581 ± $2,858; mean difference ([95% CI] $341 [$146 – $537]) expenditures.

### Factors associated with death in hospital and ICU

Factors associated with dying in hospital (Figure 1) included late referral to palliative care (OR: 2.46; 95% CI: 2.08-2.92), lower income quintile (OR: 1.24; 95% CI: 1.01-1.51), outpatient palliative care visits (OR: 1.22; 95% CI: 1.04-1.44), and undergoing renal replacement therapy during the index hospital stay (OR: 1.34; 95% CI: 1.11-1.63). Increasing age (OR: 0.93; 95% CI: 0.90-0.96), Charlson comorbidity index ≤2 (OR: 0.66; 95% CI: 0.54-0.80), residing in long-term care (OR: 0.40; 95% CI: 0.26-0.62), early (OR: 0.50; 95% CI: 0.42-0.65) or intermediate (OR: 0.52; 95% CI: 0.42-0.65) term palliative care initiation, palliative care home visits (OR: 0.14; 95% CI: 0.11-0.18), and inpatient palliative care visits (OR: 0.71; 95% CI: 0.57-0.89) were all associated with reduced odds of dying in hospitals.

Factors associated with dying in ICU included lower income quintile (OR: 1.40; 95% CI: 1.09-1.79) and late palliative care referral (OR: 1.36; 95% CI: 1.12-1.63). Older age (OR: 0.82; 95% CI: 0.79-0.85) and early (OR: 0.34; 95% CI: 0.26-0.45) and intermediate (OR: 0.33; 95% CI: 0.23-0.48) term palliative care referrals were associated with reduced odds of dying in ICU. All forms of palliative care visits were also associated with reduced odds of death in ICU (Figure 2).

## Discussion

In our population-based retrospective cohort study of AMI-CS survivors, there were high rates of admission to acute care and ICU in the last 30 days of life with a sharp upswing in rates of critical care admissions in the last days of life. First, we note that palliative care involvement was rare in patients during their index hospitalization despite a high morbidity and mortality in follow-up. Patients who utilized palliative care services in the last year of life spent increased days in acute care hospitals at end-of-life and had higher resource utilization; predominantly driven by increased inpatient cost expenditures. Early and intermediate-term referrals to palliative care occurred in a minority of patients but were associated with greatly reduced likelihood of dying in hospital and ICU. This work provides important, novel data on end-of-life trajectories and palliative care usage in survivors of AMI-CS, with important implications for patients and providers.

AHA and Canadian Cardiovascular Society guidelines both recommend palliative care consultation in the setting of advanced heart failure to improve patient-centered outcomes and quality of life.^16^^,^^26^^,^^27^ However, AMI-CS survivors—despite their similar long-term prognosis—differ from patients with acute-on-chronic heart failure.^28^ Patients with AMI-CS experience an acute and sudden deterioration in health during their index event and often have rapid improvement in symptoms with revascularization.^29^ This contrasts with the classic conceptual model of advanced heart failure,^30^ and thus may affect patient engagement with palliative care services and end-of-life care. Expansion of palliative care in cardiovascular patients remains an important AHA priority.^31^

Prior studies examining palliative care utilization in context of CS have demonstrated higher utilization of palliative care services than our current study ranging from 4.5% to 8.6% during the index hospital admission, compared to the findings of our study.^10^^,^^32^ The reasons for these differences are likely multifactorial. Prognostication during acute AMI-CS remains challenging which may delay involvement of palliative care services by the medical team until death appears certain.^33^^,^^34^ In prior studies, the majority of patients who had palliative care consultations during their index AMI-CS admission died in hospital.^10^ This explanation is particularly relevant since we excluded AMI-CS patients who died during the index hospital admission. In addition, patients may also have been receiving care that was palliative in nature by their medical teams without formal palliative care consultation.

We also examined postdischarge palliative care utilization in AMI-CS survivors and demonstrated significantly higher rates of palliative care engagement, which is a novel finding. To our knowledge, our study represents the first assessment of long-term palliative care involvement in AMI-CS patients after their index admission. Rates of palliative care utilization were even higher than a similar provincial cohort of chronic heart failure patients.^35^ One potential explanation for this finding may be due to recognition of poor prognosis by the treating clinicians. Alternatively, this may have been due to a high symptom burden in our current cohort. AMI-CS survivors remain at high risk for death, hospital readmission, and poor functional status.^5^ Our cohort of AMI-CS patients went on to die during follow-up and may thus been particularly symptomatic, potentially explaining higher than expected palliative care service utilization. Supporting this was the finding that patients who received palliative care in their last year of life were less likely to die at home and had higher number of hospital and ICU days at the end-of-life, potentially due to severe, refractory symptoms which may have required admission to acute care hospitals for management.

Despite higher-than-expected use of palliative care services, initial consultations mostly occurred late in the disease course. Late palliative referral may reflect the lack of consensus on when to integrate palliative care for patients with cardiovascular disease, particularly in the context of the numerous pharmacological, procedural, and device-based therapies available to patients living with heart failure.^36^ In contrast, among patients with cancer, palliative care is integrated earlier in the disease course and especially within the last year of life. Patients with cancer are much less likely to die in acute care hospitals and have higher number of days at home at end-of-life.^24^ While oncological guidelines exist to outline optimal referral timing, heart failure guidelines are discordant on optimal timings for involvement of palliative care.^37^^,^^38^ Further challenges arise when considering AMI-CS patients, who represent a group with particularly high short- and long-term mortality without a clear-cut transition into a terminal phase of life; as opposed to patients with cancer.^5^

Timing of palliative care referral was strongly associated with patient trajectory at end-of-life in our study. We found only early- and intermediate-term referrals were associated with a higher likelihood of dying at home. Death at home is the preferred location of death for patients with serious illness and is a key quality metric in palliative care delivery.^39^^,^^40^ In contrast, late referrals were associated with higher rates of death in hospital and ICU. Late referrals may not have allowed for sufficient time to alter patient trajectory or overall symptom burden vs earlier referrals which are associated with improved quality of life.^41^ Earlier integration of palliative care may thus be an avenue to avoid ICU admissions at end-of-life (thus reducing exposure to potentially painful and invasive interventions), improve care delivery to be congruent with patient wishes, and with minimal changes to overall resource utilization. However, further research is needed to better define this. Our findings also highlight the increasingly recognized need for cardiologists with training and expertise in provision of palliative and supportive care.^42^

Despite higher total admissions to hospital and resource utilization in patients using palliative care in the last year of life, total expenditures were only modestly higher than in those who did not use palliative care services, and this was mostly driven by higher inpatient costs. Patients with advanced heart failure and CS account for high resource utilization and care complexity when admitted to hospital. This trend is expected to further increase over time as mechanical circulatory support becomes more common with potential for reduced short-term mortality.^43^ Our findings that earlier palliative care integration was associated with avoidance of costly and resource intensive admissions at end-of-life may serve as a means to optimize high-quality and cost-effective care in this patient population.

### Study Limitations

This study has some important limitations. First, due to methodological challenges inherent to our population-based analysis, we were unable to determine causes for hospital and ICU admission near end-of-life, nor cause of death. We were thus unable to quantify the cardiovascular vs non-cardiovascular burden of illness in AMI-CS survivors. Second, our definition of palliative care relied on capturing administrative billing codes, capturing a heterogeneous mixture of specialty and primary palliative care. We thus could not determine the quality of the palliative care services that patients received, only the date of initiation. Third, we were unable to disentangle the timing of palliative care receipt from, for example, the timing hospital admissions or other end-of-life health care utilization. This requires much more complex modeling to account for the time-varying nature of health care utilization at the end of life to derive valuable insights. We instead modeled death in hospital/ICU since it is a terminal event, so the explanatory variables must have occurred before. Future research will need to disentangle more granular temporal sequences between palliative care receipt and nonterminal events such as a hospital admission or other aggressive end of life interventions. There remains potential for reverse causation and other forms of bias given the time-varying exposures within the study. The association between palliative care timing and outcomes should not be interpreted as causal. Fourth, due to the administrative nature of the data set, we could not identify important patient characteristics such as frailty, nor quantify important patient-centered outcomes such as self-reported quality of life, symptom burden, patient preferred place of death, nor reasons for palliative care referral. Instead, there was a focus on identifying important patient-centered outcomes such as place of death.^44^^,^^45^ Future qualitative studies are required to further assess the patient experience of AMI-CS survivors. Due to the inability to capture such important clinical details, there is the potential for residual confounding. Fifth, we attempted to account for geographical differences in patient care and outcome by including rurality and neighborhood income quintile along with the patient factors. However, future research should investigate variation across more granular geographic distributions and accounting for health care access.

Finally, our findings may lack generalizability to other health care systems with other palliative care infrastructure. Ontario has a universal health care system with a relatively higher hospital admissions at end of life and a different palliative care structure compared to other jurisdictions.^46^ Outcomes such as ICU and in-hospital death are conditional on care pathways within health care systems, which may influence our observed associations independent of underlying patient risk. Our findings thus require replication in other health care settings.

## Conclusions

In this descriptive, population-based analysis of survivors of AMI-CS, early palliative care involvement was low despite high burden of morbidity and mortality in follow-up. While the majority of patients received palliative care in the final year of life, palliative care was most commonly initiated in the last 14 days of life. In contrast, early and intermediate term palliative care involvement was associated with important patient-centered outcomes of reduced risk of death in hospital and ICU, and more time in the community. Palliative care utilization was associated with higher resource utilization. Formal guidelines on timing of palliative care involvement are needed in patients surviving AMI-CS.

Clinical Perspectives **COMPETENCE IN MEDICAL KNOWLEDGE:** There were high rates of admission to acute care and ICU in the last 30 days of life, with a sharp upswing in rates of critical care admissions in the last days of life. Early and intermediate-term referrals to palliative care occurred in a minority of patients but were associated with a greatly reduced likelihood of dying in hospital and ICU.

**TRANSLATIONAL OUTLOOK:** In the current data set, it is challenging to disentangle the timing of palliative care receipt from, for example, the timing hospital admissions or other end-of-life health care utilization. Future research will need to disentangle more granular temporal sequences between palliative care receipt and nonterminal events such as a hospital admission or other aggressive end of life interventions.

## Funding support and author disclosures

This study was supported by ICES, which is funded by an annual grant from the Ontario 10.13039/501100000226Ministry of Health (MOH) and Ministry of Long-term Care (MLTC). The opinions, results, and conclusions reported in this paper are those of the authors and are independent from the funding sources. No endorsement by ICES or the Ontario 10.13039/501100000226MOH/MLTC is intended or should be inferred. Parts of this material are based on data and/or information compiled and provided by the 10.13039/100030898Canadian Institute for Health Information (CIHI). However, the analyses, conclusions, and opinions and statements expressed in the material are those of the authors, and not necessarily those of CIHI. This document used data adapted from the Statistics Canada Postal CodeOM Conversion File, which is based on data licensed from Canada Post Corporation, and/or data adapted from the Ontario Ministry of Health Postal Code Conversion File, which contains data copied under license from ©Canada Post Corporation and Statistics Canada. This study is also supported by the Innovation Fund of the Alternative Funding Plan for Academic Health Sciences Centres of Ontario. Dr Brodie previously consulted for LivaNova; has been on the medical advisory boards for Medtronic, Inspira, Cellenkos, HBOX Therapies, and Vantive; is the President of the Extracorporeal Life Support Organization (ELSO) and the Chair of the Board of the International ECMO Network (ECMONet); and writes for UpToDate. Dr Fan reports personal fees from Aerogen, Boehringer Ingelheim, Getinge, HBOX Therapies, Inspira, Mallinckrodt, Vantive, and Vasomune outside the submitted work. All other authors have reported that they have no relationships relevant to the contents of this paper to disclose.
