# Supplementary material for: Palliative and End-of-Life Care Utilization in Cardiogenic Shock Complicating Acute Myocardial Infarction: A Population-Based Study
Source: JACC Adv. 2026 Jun 11;5(7):102869. doi: 10.1016/j.jacadv.2026.102869 (PMC13276325; doi:10.1016/j.jacadv.2026.102869)
Supplement: Supplemental Material [file mmc1.pdf]

Table S1. Databases linked through ICES, on the basis of individual patient identifiers.

Database	Information Contained
The Ontario Health Insurance Plan (OHIP) Claims Database	All data on physician fee-for-service claims for inpatient and outpatient services.
Canadian Institute for Health Information (CIHI) Discharge Abstract Database (DAD)	Data from all acute care hospitalizations, including detailed diagnostic and procedural information.
Registered Persons Database	Deaths and all demographic information.
Home Care Database	Publicly-funded homecare services.
National Ambulatory Care Reporting System (NACRS)	Data related to all Emergency Department use.
National Rehabilitation Reporting System	Data related to all inpatient rehabilitation programs.
Continuing Care Reporting System	Data on long-term care (e.g., nursing home) and complex continuing care (e.g., rehabilitation facility) use.
Statistics Canada Census	Data related to income quintile and rurality, through postal codes
Vital Statistics Database: The Office of the Registrar General – Deaths	Information on cause and date of death

Table S2. Relevant databases and codes utilized for key variables

Definition	**Associated database and codes**
Invasive mechanical ventilation	Captured via DAD Intervention code falling between admission/discharge of hospitalization:<br>1GZ31CAEP<br>1GZ31CAND<br>1GZ31CAPK<br>1GZ31CRND<br>1GZ31GPND<br>1GZ31JAGX
Renal replacement therapy	Database: Canadian Organ Replacement Register<br>Codes:<br>In RECIPIENT_ TREATMENT database, select all chronic dialysis patients with valid IKN<br>[Treatment_Code not equal to "171", "181"]<br><br>Database: Discharge Abstract Database Codes:<br>ICD9: V451, V560, V568<br>ICD10: Z49, Z992<br><br>CCP: 5195, 6698 CCI: 1PZ21<br><br>Database: Ontario Health Insurance Plan<br>Codes:<br>R849, G323, G325, G326, G860, G862, G865, G863, G866, G330, G331, G332, G333, G861, G082, G083, G085, G090, G091, G092, G093, G094, G095, G096, G294, G295, G864, H540, H740
**Invasive Coronary Angiography**	INCODE: 3IP10
Intra-aortic balloon pump	Database: Critical Care Information System<br>Codes:<br>"INTRAAORTICBALLOONPUMP"<br><br>AND OHIP Z743 or Z780
Impella	(INCODE: 1HP53GPQP**)**
ECMO	DAD_INCODE:<br>1LZ37GPQM (installation)<br>1LZ37HHGB (installation)<br>1LZ37LAQM (installation)<br>1LZ37GPGB (installation)<br>1LZ38JAGB (management)<br>1LZ38JAGC (management)<br>1LZ38JAQM (management)<br><br>AND

	OHIP_FEECODE: Z788 (ECMO physician billing flag)<br><br>A given hospital episode must have both an INCODE recorded and Z788 billed where the servate occurred between the episode admission date and discharge date +/- 3 days on each side.

Abbreviations: Abbrevations: ECMO = Extracorporeal Membranous Oxygenation, ICD= International Statistical Classification of Diseases and Related Health Problems, CCI = Canadian Classification of Health Interventions, IKN = ICES Key Number, INCODE = Intervention Code, CCP = Canadian Classification of Diagnostic, Therapeutic, and Surgical Procedures

All available health administrative data (OHIP, DAD, ODB) during the index was used to ascertain specific ICU interventions and based on previously published algorithms/ ICES cohorts (see below, respectively)

1. Fernando SM, Qureshi D, Tanuseputro P, Talarico R, Hibbert B, Mathew R, Rochwerg B, Belley-Côté EP, Fan E, Combes A, Brodie D, Schmidt M, Simard T, Di Santo P, Kyeremanteng K. Long-term mortality and costs following use of Impella® for mechanical circulatory support: a population-based cohort study. Can J Anaesth. 2020 Dec;67(12):1728-1737. English. doi: 10.1007/s12630-020-01755-9. Epub 2020 Jul 15. PMID: 32671805.

2. Fernando SM, Qureshi D, Tanuseputro P, Fan E, Munshi L, Rochwerg B, Talarico R, Scales DC, Brodie D, Dhanani S, Guerguerian AM, Shemie SD, Thavorn K, Kyeremanteng K. Mortality and costs following extracorporeal membrane oxygenation in critically ill adults: a population-based cohort study. Intensive Care Med. 2019 Nov;45(11):1580-1589. doi: 10.1007/s00134-019-05766-z. Epub 2019 Sep 16. PMID: 31529353.

Table S3 Ontario Health Insurance Plan (OHIP), International Classification of Diseases, Version 9 (ICD-9), and Version 10 (ICD-10) diagnostic codes for categorization of comorbidities.

**Condition [reference for validated algorithm where applicable]**	**ICD9/OHIP**	**ICD10**
Acute Myocardial Infarction[1]	410	I21, I22
Atrial Fibrillation/Flutter	42731, 42732	I480, I481
Cancer	140-239	C00-C26, C30-C44, C45- C97
Chronic Kidney Disease	403, 404, 584, 585, 586, v451	N17, N18, N19, T82.4, Z49.2, Z99.2
Chronic Obstructive Pulmonary Disease[2]	491, 492, 496	J41, J43, J44
Cirrhosis[3]		
Congestive Heart Failure[4]	428	I500, I501, I509
Diabetes Mellitus[5]	250	E08-E13
Dyslipidemia[6]	272	E78.0, E78.2, E78.4, E78.5
Hypertension[7]	401, 402, 403, 404, 405	I10, I11, I12, I13, I15
Stroke	430, 431, 434, 436	I60, I62, I63, I64
Prior CABG	CCI: 1IJ76	
Prior PCI:	CCI: 1IJ50, 1IJ54, 1IJ57GQ	
STEMI		I21, I22 and with secondary R9430

Abbreviations: CCI = Canadian Classification of Health Interventions DAD=Discharge Abstract Database; ICD = International Classification of Disease; ODB = Ontario Drug Benefit Claims database; OHIP = Ontario Health Insurance Plan Claims Database
All available health administrative data (OHIP, DAD, ODB) prior to index is used to ascertain disease status
COPD, CHF, Dementia, Diabetes, Hypertension, Previous Major Hemorrhage are based on validated case algorithms/ ICES cohorts (see below, respectively)

References:

1. Ko DT, Ahmed T, Austin PC, Cantor WJ, Dorian P, Goldfarb M, Gong Y, Graham MM, Gu J, Hawkins NM, Huynh T, Humphries KH, Koh M, Lamarche Y, Lambert LJ, Lawler PR, Légaré JF, Ly HQ, Qiu F, Quraishi AUR, So DY, Welsh RC, Wijeysundera HC, Wong G, Yan AT, Gurevich Y. Development of Acute Myocardial Infarction Mortality and Readmission Models for Public Reporting on Hospital Performance in Canada. CJC Open. 2021 May 1;3(8):1051-1059. doi: 10.1016/j.cjco.2021.04.012. PMID: 34505045; PMCID: PMC8413230.
2. Gershon AS, Wang C, Guan J, Vasilevska-Ristovska J, Cicutto L, To T. Identifying Individuals with Physician Diagnosed COPD in Health Administrative Databases. Copd 2009;6:388–94.
3. Lapointe-Shaw L, Georgie F, Carlone D, Cerocchi O, Chung H, Dewit Y, Feld JJ, Holder L, Kwong JC, Sander B, Flemming JA. Identifying cirrhosis, decompensated cirrhosis and hepatocellular carcinoma in health administrative data: A validation study. PLoS One. 2018 Aug 22;13(8):e0201120. doi: 10.1371/journal.pone.0201120. PMID: 30133446; PMCID: PMC6104931.
4. Schultz SE, Rothwell DM, Chen Z, Tu K. Identifying cases of congestive heart failure from administrative data: a validation study using primary care patient records. Chronic Diseases and Injuries in Canada 2013;33:160–6.
5. Hux JE, Ivis F, Flintoft V, Bica A. Diabetes in Ontario: Determination of prevalence and incidence using a validated administrative data algorithm. Diabetes Care 2002;25:512–6.
6. Marrie RA, Yu BN, Leung S, Elliott L, Caetano P, Warren S, Wolfson C, Patten SB, Svenson LW, Tremlett H, Fisk J, Blanchard JF; CIHR Team in Epidemiology and Impact of Comorbidity on Multiple Sclerosis. Rising prevalence of vascular comorbidities in multiple sclerosis: validation of administrative definitions for diabetes, hypertension, and hyperlipidemia. Mult Scler. 2012 Sep;18(9):1310-9. doi: 10.1177/1352458512437814. Epub 2012 Feb 10. PMID: 22328682.
7. Tu K, Campbell NR, Chen ZL, Cauch-Dudek KJ, McAlister FA. Accuracy of administrative databases in identifying patients with hypertension. Open Med 2007;1:e18–26.

Figure S1. Kaplan-Meier Survival Curve of Cardiogenic Shock complicating Acute Myocardial Infarction Survivors who died in follow-up

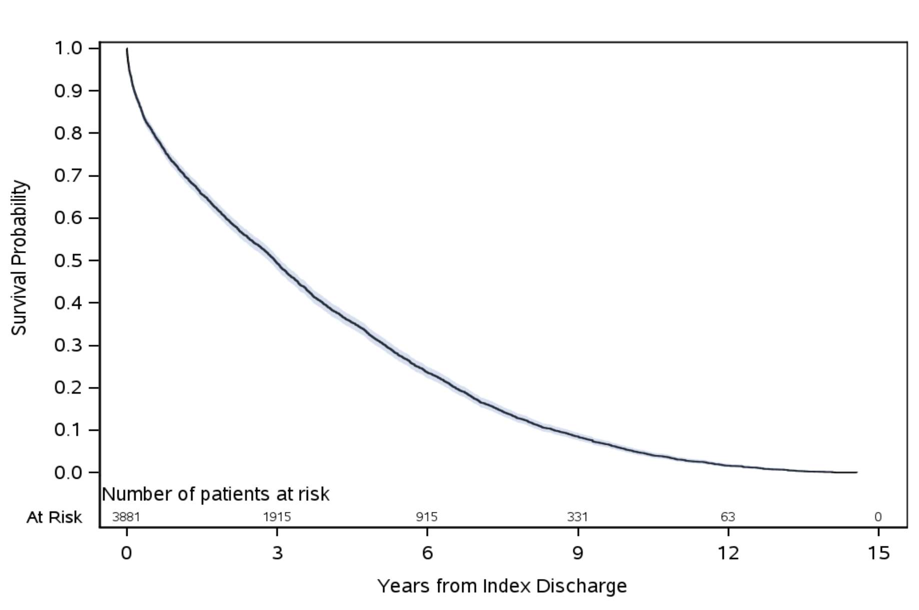

Deleted: ***Figure S1***

Figure Legends

Figure S1. Kaplan-Meier Survival Curve

Kaplan-Meier Survival Curve of Survivors of Cardiogenic Shock complicating Acute Myocardial Infarction Survivors who Proceeded to Die During Follow-Up

Formatted: Font: Not Italic, Underline

Formatted: Underline

Formatted: Font: Not Bold, Not Italic

Formatted: Font: Not Bold, Not Italic

Formatted: Font: Not Bold, Not Italic

Formatted: Font: Not Bold, Not Italic

Formatted: Font: Not Bold, Not Italic

Formatted: Font: Not Bold, Not Italic
